# Patterns of Physical Activity Across the Retirement Transition in Older Adults

**DOI:** 10.1093/geroni/igaf122.2907

**Published:** 2025-12-31

**Authors:** Hyesu Yeo, Michelle Gray

**Affiliations:** University of Arkansas, Fayetteville, Arkansas, United States; University of Arkansas, Fayetteville, Arkansas, United States

## Abstract

Purpose Many studies highlight the health benefits of retirement and its positive relationship with physical activity (PA) and cognition. To better understand this interaction, this study examined PA patterns and their associations with physical health, mental health, and cognition across the retirement transition. Methods Using data from the 2006–2018 RAND Health and Retirement Survey (HRS) and the 2007–2017 HRS Consumption and Activities Mail Survey, this study analyzed 91 retirees aged 60 to 80 (226 non-retirees as a reference group). Graphical analyses compared retirees and non-retirees across eight PA types adapted from the International Classification of Functioning. Bivariate and correlation analyses examined associations between PA, health, cognition, and demographics across pre-retirement and post-retirement. Results After retirement, PA in personal care, caring for housework, social activities, and arts & crafts significantly increased. Most PA declined pre-retirement but showed a rebound at retirement, except for solitary activities, which continued to decline. In post-retirement, PA levels decreased again. PA patterns among non-retirees remained stable. Education(ρ = 0.36), women(ρ ≈ 2.64-2.89), White race(U ≈ 2.43-2.28), urban living (U = -2.44), mental health(ρ = 0.28), and cognition(ρ ≈ 0.24-0.39) were positively related to specific PA types at retirement, which, in turn, positively influenced mental health(ρ ≈ 0.27-0.32) and cognition(ρ ≈ 0.21-0.34) post-retirement. Conclusion This study found a PA decline before retirement, an increase at retirement, and a later decrease, mirroring changes in mental health and cognition. Interventions should aim to prevent PA decline pre-retirement and support sustained PA during the transition into retirement while considering individual differences.

